# Pilot Study on the Effects of a Cosmetic Serum Containing Niacinamide, Postbiotics and Peptides on Facial Skin in Healthy Participants: A Randomized Controlled Trial

**DOI:** 10.3390/life14121677

**Published:** 2024-12-18

**Authors:** Doris Rusic, Magdalena Ivic, Ana Slugan, Dario Leskur, Darko Modun, Toni Durdov, Dubravka Vukovic, Josipa Bukic, Josko Bozic, Ana Seselja Perisin

**Affiliations:** 1 Department of Pharmacy, University of Split School of Medicine, 21000 Split, Croatia; drusic@mefst.hr (D.R.); magdalena.ivic16@gmail.com (M.I.); ana.slugan2@gmail.com (A.S.); dleskur@mefst.hr (D.L.); dmodun@mefst.hr (D.M.); toni.durdov@mefst.hr (T.D.); aperisin@mefst.hr (A.S.P.); 2 Department of Dermatovenerology, University Hospital Split, 21000 Split, Croatia; dvukovic@mefst.hr; 3 Department of Laboratory Medicine and Pharmacy, Faculty of Medicine, Josip Juraj Strossmayer University of Osijek, 31000 Osijek, Croatia; 4 Department of Pathophysiology, University of Split School of Medicine, 21000 Split, Croatia; jbozic@mefst.hr

**Keywords:** niacinamide, skin hydration, transepidermal water loss, skin erythema, peptides, postbiotics

## Abstract

The literature describes niacinamide, but also postbiotics and peptides, as ingredients that improve skin barrier function, but also affect melanin and sebum levels in individuals. However, the combined effects of these ingredients in a single cosmetic serum have not been sufficiently investigated. Therefore, the aim of this randomized controlled study, conducted at the University of Split School of Medicine (Croatia), was to evaluate the effects of cosmetic products containing these active ingredients. This study was registered with ClinicalTrials.gov (NCT06189105) and has been completed. Primary outcomes were the levels of trans epidermal water loss, skin hydration, erythema, melanin, and sebum, all measured in 25 healthy Caucasian participants. Significant differences between hydration levels were observed at week 4 (61.0 ± 11.2 vs. 68.6 ± 13.3 AU, control and intervention). Moreover, a significant decrease in erythema values from the first to last measurement in the intervention group was observed, (379.9 ± 106.8 vs. 333.6 ± 73.5 AU, baseline values and week 4, intervention group). Interestingly, both the increase in skin hydration levels and the decrease in skin erythema after niacinamide serum application were significant in study participants who did not use sun protection products. It is well known that ultraviolet radiation has detrimental effects on human skin, and our results suggest that niacinamide could help counteract these effects.

## 1. Introduction

The skin is the outermost layer that surrounds our body and serves as a defense against external aggressors. It acts as a protective barrier and plays a crucial role in maintaining homeostasis by minimizing water loss and helping regulate the body’s temperature [1]. Skin is also the most visible organ, and its health is connected to the general well-being of individuals. Furthermore, skin health is significant from a medical as well as an aesthetic perspective [2]. The aesthetic aspect of skin has led to the development of numerous cosmetic ingredients which are available to consumers as serums, lotions, creams and ointments and are now seen as an appealing alternative to some more invasive cosmetic treatments [3].

In recent decades, postbiotics and peptides, in addition to other ingredients, have gained popularity among consumers. Peptides, polymers of amino acids, are widely used as topical ingredients for the signs of aging skin. Currently, four groups of peptides are described in the literature: peptides that trigger collagen synthesis, peptides which inhibit enzymes, carrier peptides, and peptides which inhibit neurotransmitters. In the last 20 years, many new peptides, used for cosmetic purposes, have been developed [4]. Postbiotics, byproduct compounds produced by bacteria, have gained popularity in cosmetic products since it is assumed they may improve the composition of the skin microbiome. It has been established that the skin microbiome is involved in maintaining healthy skin. Key components under the name postbiotics are cell-free supernatants, lysates and bioactive peptides [5].

To our knowledge, the most diverse ingredient used in the cosmetics industry is niacinamide, which has a variety of mechanisms of action documented in the literature [6,7,8]. For example, it has been established that niacinamide enhances the synthesis of ceramide, through the expression of serine palmitoyl transferase, an enzyme involved in the synthesis of sphingolipids. Since niacinamide also accelerates keratinocyte differentiation and prevents their senescence caused by oxidative stress, this ingredient is widely used to strengthen the skin barrier [9]. Niacinamide has been studied in the field of dermatology concerning its use in patients with acne vulgaris and psoriasis, as well as the prevention of skin cancer [10,11,12,13]. Niacinamide has also been studied in cosmetics for its ability to reduce inflammation, decrease skin pigmentation, promote anti-aging, and boost intercellular lipid production, which improves the barrier function of the stratum corneum [14,15].

To our knowledge, while previous studies have individually examined niacinamide, postbiotics, and peptides, the combined effects of these ingredients in a single cosmetic serum have not been sufficiently investigated. Therefore, the aim of this research was to investigate the effects of niacinamide, postbiotics and peptides cosmetic serum. Primary outcomes were skin barrier measurements, melanin and sebum levels.

## 2. Materials and Methods

The trial was registered on clinicaltrials.gov under study ID NCT06189105. The design of this study was a randomized controlled clinical trial. It was conducted at the University of Split School of Medicine in May and June 2023. The Ethics Committee of the University of Split School of Medicine approved the study, and it was conducted in accordance with ethical principles.

The study involved 25 healthy male and female participants, Fitzpatrick II–III phototype, aged 21 to 29 years. None of the participants had a history of skin diseases. All participants gave informed consent prior to the study, in which the objectives, methods, and procedures of the research were detailed. The study had specific exclusion criteria, which included the absence of skin diseases, skin cancer, or sun damage in the target area of the research, as well as abstaining from using immunomodulators, corticosteroids, or antihistamines for 30 days before the tests. Additionally, participants had to adhere to the study protocol. Other exclusion criteria included significant exposure to natural or artificial ultraviolet radiation, pregnancy, breastfeeding, a history of vitiligo, melasma, or other hyperpigmentation/photosensitivity disorders, immunosuppression, and allergies to any components of the serum used in the intervention. Before the study, participants were also asked about any previous adverse reactions to the serum ingredients.

The forehead of the participants was chosen as the test site for the study. Using the Excel^®^ software program (version 16.0, Microsoft Corporation, SAD, Redmond, DC, USA), randomization determined which side of the forehead would receive the niacinamide serum and which side would remain untreated, following the usual routine. The usual routine included only hydrating cream, and any other active ingredients were prohibited during the study period (e.g., retinol, acids etc.). After measuring the baseline skin parameters, participants were instructed on how to apply the serum. Each participant received one sample of the serum. The hydrating serum, commercially available under the name Face it you love me (Miss Alice, Ljubljana, Slovenia), with niacinamide, is formulated for problematic and/or sensitive skin. The key ingredients of the serum are niacinamide, hyaluronic acid, cranberry extract, Sr-spider Polypeptide-1 and postbiotic leuconostoc/radish root ferment lysate filtrate. Full ingredients list is available as Supplementary Materials. The serum was applied once daily to the designated area in the evening before bed, using three drops. On the measurement days, participants were instructed not to use any cosmetics on the tested area. The serum was weighed before the start and after the end of the study to confirm adherence to the protocol.

Five skin parameters were measured using a non-invasive bioengineering method with the Multi Probe Adapter 6 device (Courage+Khazaka GmBH, Cologne, Germany). Skin barrier function was determined by measuring transepidermal water loss (TEWL). The Multi Probe Adapter 6 device consists of Tewameter^®^ TM 300, Corneometer^®^ CM 825, Mexameter^®^ MX 18, Sebumeter^®^ SM 815. The Tewameter^®^ TM 300 assesses TEWL by calculating the evaporation rate and expresses the results in g/m^2^/h [16]. Skin hydration was evaluated using the Corneometer^®^ CM 825, which operates based on capacitance measurements. The probe converts the skin’s capacitance values into arbitrary hydration units ranging from 0 to 120 [17]. Skin erythema and melanin content were measured with the Mexameter^®^ MX 18 probe. This probe measures the absorption and reflection of light. Green and red light are particularly suitable for erythema assessment as they correspond to hemoglobin’s absorption spectrum, while the melanin index is calculated using the absorption of red and infrared light [18]. The Sebumeter^®^ SM 815 was used to measure sebum levels on the skin surface. This probe utilizes a transparent film that absorbs sebum from the skin. The light transmission through the film is measured before and after contact with the skin using a photometer, providing an accurate quantification of sebum in µg/cm^2^ [19].The conditions in the measurement room were kept constant. The room temperature was maintained between twenty and twenty-two degrees Celsius. Air humidity was controlled in the range of 40 to 60 percent using a humidifier (Gorenje, Velenje, Slovenia). Before the measurements, participants were instructed to spend 15 min in the room to acclimatize to the conditions.

After the objective assessment of skin parameters using the probes, participants were asked about their personal experiences with the serum, including their subjective perceptions and any potential side effects such as itching, irritation, etc. The total duration of the study was 5 weeks, with a total of five measurements conducted. Baseline values of skin parameters were measured on the first day, and the intervention was introduced. On the eighth, fifteenth, twenty-second, twenty-ninth, and thirty-sixth days, skin values on both sides were measured. Each day, from the measurement of baseline values, the serum was applied to the designated area in the evening before bedtime.

Participants’ demographic characteristics are presented in Table 1. The most represented age was 24-years-old (9 out of 25 participants). Most of the participants were female (80%) and declared as non-smokers (76%). More than half of the participants stated that they had not used sun protection cream every day (60%) with the rest using it constantly. Participants were asked about their skin type with the following distribution: seven of them had an oily skin type, six of them had combination skin type, the same number as dry skin type and normal skin type.

Statistical analysis was performed using IBM SPSS Statistics software (version 25). Two-Way Repeated Measures ANOVA was employed to compare changes over time between the two groups. The total change over the study period was calculated by subtracting the baseline value from the final measurement value. An Independent Samples *t*-Test was used to compare the means of the two groups at each specific time point and to compare the means of the total change between the two groups. A Paired Samples *t*-Test was used to compare values within the same group at the beginning and end of the study. Statistical significance was determined at *p* < 0.05. Data are presented as mean ± standard deviation, unless otherwise specified.

## 3. Results

**Figure 1:**
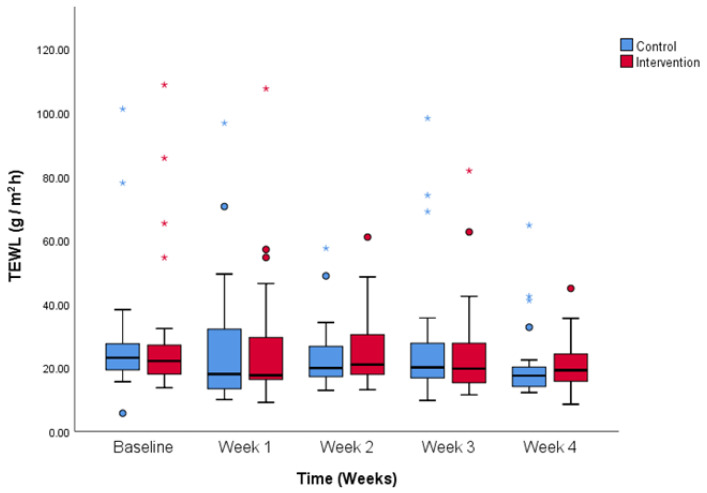
TEWL values over the period of the study. Comparison between control and intervention group. Figure shows no significant differences between control and intervention group throughout the study. Colored dots show less extreme outliers and colored stars more extreme outliers.

Baseline TEWL values were comparable between the groups (28.3 ± 20.0 vs. 30.4 ± 23.6 g/m^2^h, control and intervention). TEWL values decreased in both groups over time, as time had a significant effect on TEWL values (*p* < 0.05). Figure 1 shows there was no difference between the groups at the end of the study (21.0 ± 12.1 vs. 20.2 ± 7.8 g/m^2^h, control and intervention).

**Figure 2:**
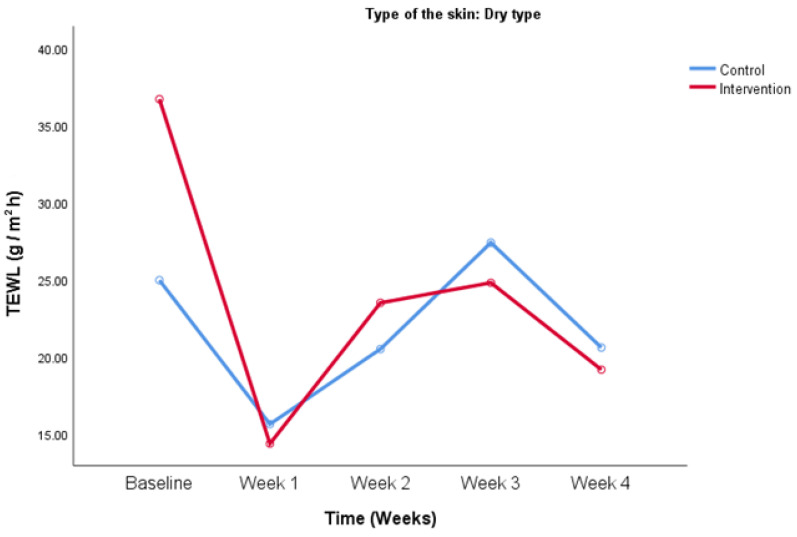
TEWL values change over the period of the study. Comparison between control and intervention group for the participants with the dry skin type.

Participants with the dry skin type had higher baseline TEWL values in the intervention group compared to the control group (24.9 ± 7.0 vs. 36.7 ± 28.1 g/m^2^h, control and intervention) although that difference was not significant. Towards the end of the study, TEWL values in both the intervention and control group approximated for the participants with the dry skin type as seen in Figure 2 (20.6 ± 10.5 vs. 19.1 ± 4.3 g/m^2^h, control and intervention on week 4).

**Figure 3:**
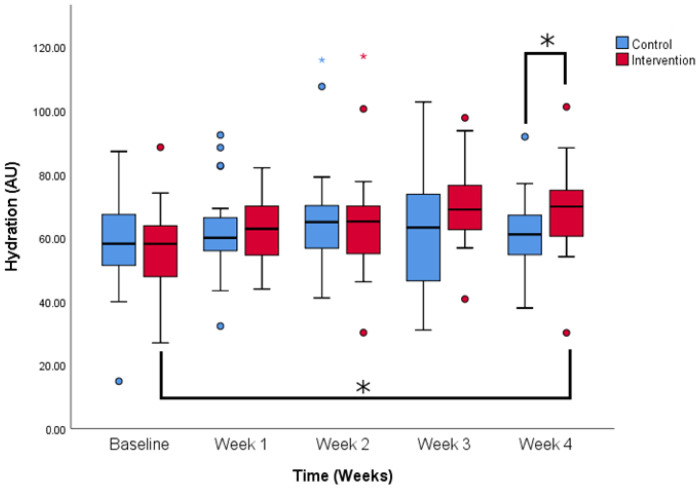
Hydration values change over the period of the study. Comparison between control and intervention group. * *p* < 0.05 (Two-way ANOVA for repeated measures with Bonferroni post-hoc test). Colored dots show less extreme outliers and colored stars more extreme outliers.

Hydration values were comparable at the beginning of the study (58.5 ± 14.9 vs. 56.4 ± 13.9 AU, control and intervention). Hydration values rose over time in both groups and the effect of time on hydration values is significant (*p* < 0.05). However, as seen in Figure 3, there is a significant difference between control and intervention at week 4 (61.0 ± 11.2 vs. 68.6 ± 13.3 AU, control and intervention). Participants who were using the tested serum had more hydrated skin at the end of the study (*p* < 0.05). Furthermore, there is a difference between the baseline value and the last measurement in the intervention group (*p* < 0.05) and there is no such difference in the control group.

**Figure 4:**
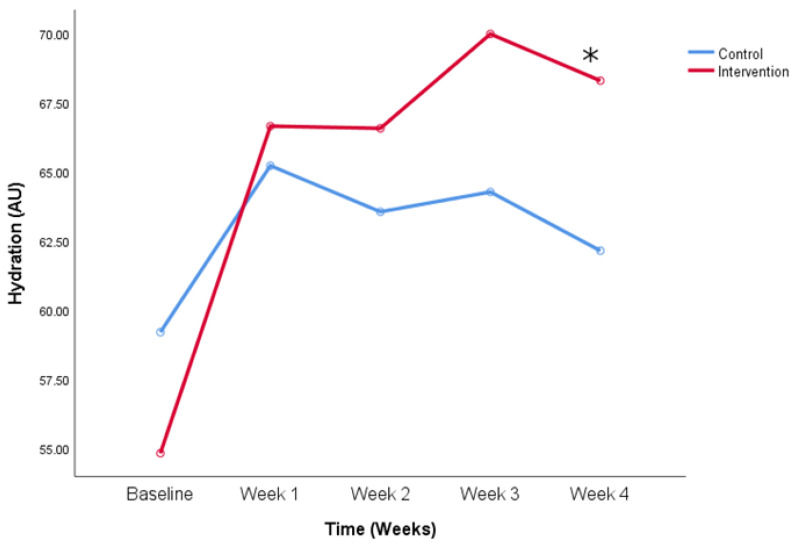
Hydration values change over the period of the study. Comparison between the control and intervention group for participants who did not use sun protection cream constantly. * *p* < 0.05 (Two-way ANOVA for repeated measures with Bonferroni post-hoc test).

Figure 4 highlights the difference in hydration values between the baseline value and last measurement, which was found in participants who did not use sun protection cream constantly (54.8 ± 14.8 vs. 68.3 ± 7.0 AU, baseline value and week 4, intervention group, *p* < 0.05) and there was no such difference in those participants who do use it, when talking about the intervention group.

**Figure 5:**
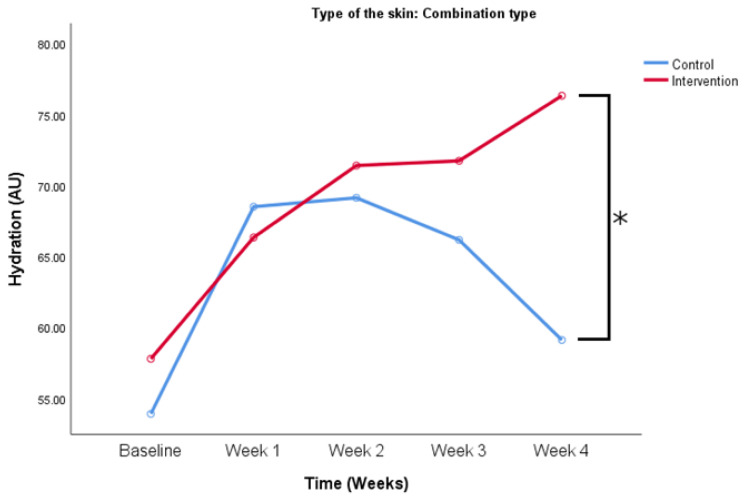
Hydration values changed over the period of the study. Comparison between control and intervention group for participants who have combination skin type. * *p* < 0.05 (Two-way ANOVA for repeated measures with Bonferroni post-hoc test).

When comparing baseline values (53.9 ± 23.2 vs. 57.8 ± 16.1 AU, control and intervention) there was no difference between groups for the participants with the combination skin type. The difference was seen in week 4 (59.1 ± 13.3 vs. 76.3 ± 12.9 AU, control and intervention), with the intervention group having higher hydration values than the control group as seen in Figure 5 (*p* < 0.05).

**Figure 6:**
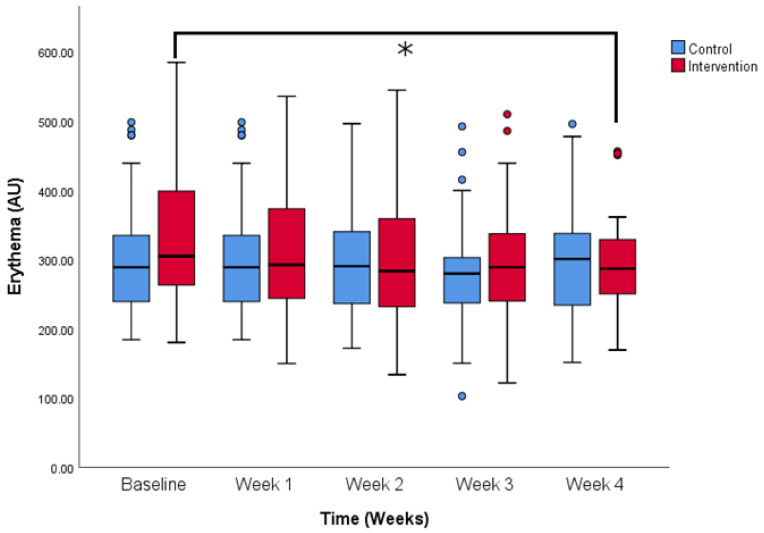
Erythema values change over the period of the study. Comparison between control and intervention group. * *p* < 0.05 (Two-way ANOVA for repeated measures with Bonferroni post-hoc test). Colored dots show less extreme outliers and colored stars more extreme outliers.

Baseline erythema values were slightly higher in the intervention group when compared to the control group, but the difference was not significant (310.5 ± 95.5 vs. 333.8 ± 107.9 AU, control and intervention). While values at the end of the study were comparable, there was a significant change between baseline erythema values and the last measurement on week 4 (333.8 ± 107.9 vs. 297.4 ± 77.4 AU, baseline values and week 4, intervention group). Erythema values decreased significantly for the group using the tested serum as Figure 6 describes (*p* < 0.05). There is no significant difference between any of the time points for the control group. Also, multivariate tests in ANOVA have shown that there is a significant effect of the intervention on the erythema values (Wilks’ Lambda test, *p* < 0.05) and the same test is insignificant in the control group.

**Figure 7:**
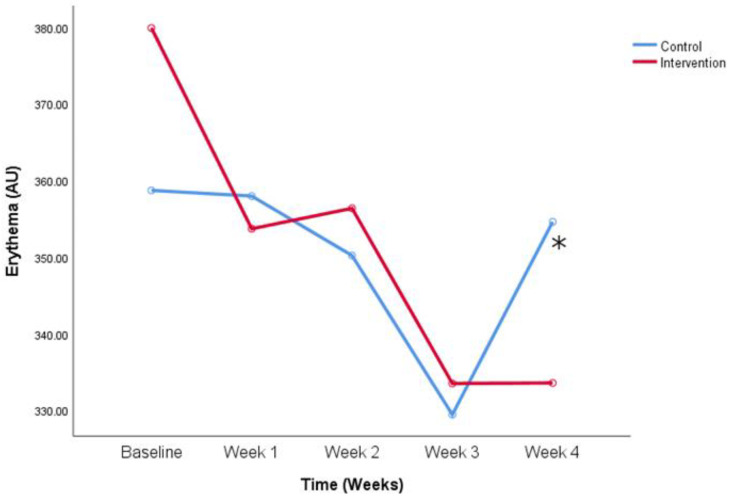
Erythema values change over the period of the study. Comparison between control and intervention group for participants who did not use sun protection cream constantly. * *p* < 0.05 (Two-way ANOVA for repeated measures with Bonferroni post-hoc test).

When conducting the same comparison in participants who did not use sun protection constantly, similar results were found. There is a significant decrease in erythema values from the first to last measurement in the intervention group (379.9 ± 106.8 vs. 333.6 ± 73.5 AU, baseline values and week 4, intervention group) and there is no such difference in the control group, for patients who do not use sun protection cream constantly. Erythema improved over time in the intervention group for the patients who did not use sun protection, as seen in Figure 7 (*p* < 0.05). There was no difference between the first and last measurement in the intervention group for the participants who use sun protection every day.

**Figure 8:**
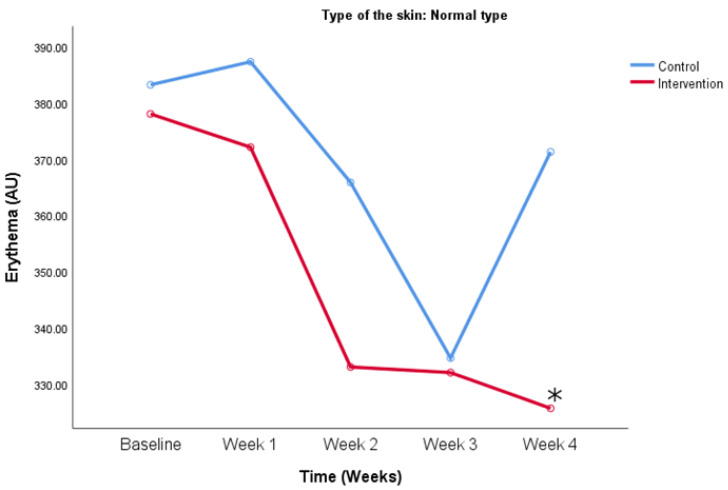
Erythema values change over the period of the study. Comparison between control and intervention group for participants who have normal skin type. * *p* < 0.05 (Two-way ANOVA for repeated measures with Bonferroni post-hoc test).

Figure 8 shows that baseline values were comparable between control and intervention group for participants with normal skin type (383.2 ± 97.3 vs. 378.0 ± 104.4 AU, control and intervention, participants with normal skin type). Erythema values decreased significantly throughout the period of the study for the intervention group (378.0 ± 104.4 vs. 325.7 ± 78.9, baseline value and week 4, participants with normal skin type, *p* < 0.05). There is no difference between the first and least measurement in the control group for the participants with the same skin type.

**Figure 9:**
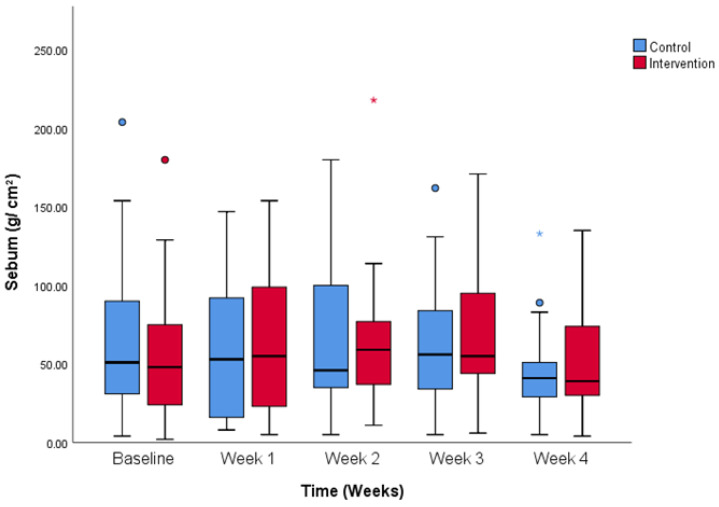
Sebum values change over the period of the study. Comparison between control and intervention group. Colored dots show less extreme outliers and colored stars more extreme outliers.

There is no significant difference in sebum values between the control and intervention group at the beginning of the study (63.2 ± 49.7 vs. 52.0 ± 41.1 g/cm^2^, control and intervention), nor at the end of the study (43.9 ± 28.2 vs. 50.4 ± 32.4 g g/cm^2^, control and intervention). Figure 9 shows that the change in sebum value is comparable between the groups across the study. The effect of time on value change is significant in both groups (*p* < 0.05).

**Figure 10:**
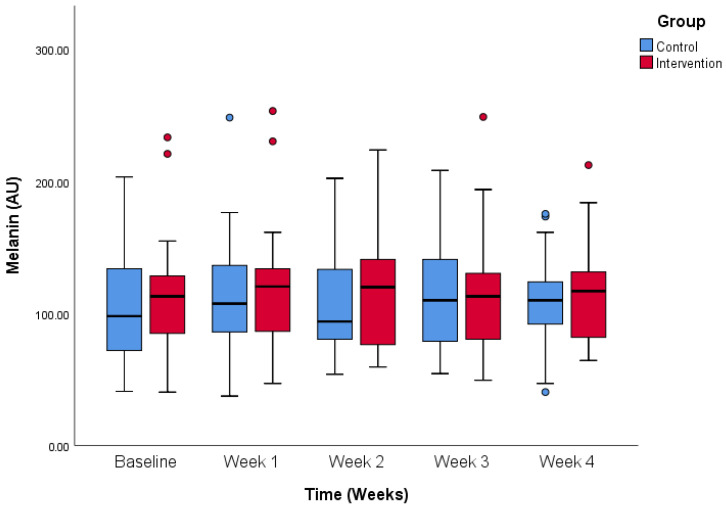
Melanin values change over the period of the study. Comparison between control and intervention group.

Melanin values were comparable between the groups over the period of the study as Figure 10 shows. Melanin values did not differ between the first (107.3 ± 41.6 vs. 113.8 ± 43.9 AU, control and intervention) and last measurement (109.5 ± 35.0 vs. 115.4 ± 39.9 AU, control and intervention).

## 4. Discussion

To our knowledge, this is one of the first studies examining effects of cosmetic formulation with niacinamide, peptide Sr-spider Polypeptide-1, and postbiotic leuconostoc/radish root ferment lysate filtrate. Based on objective measurements and subjective reports of the study participants, it can be concluded that this combination is safe for use as adverse reactions were not reported during the study period. Compared to placebo, intervention showed statistically significant differences in two measurements: skin hydration and erythema. Since niacinamide concentration in tested formulation is 5%, it can be assumed that this combination is appropriate for sensitive skin, which could react to a higher concentration of this ingredient. However, future studies should evaluate possible synergistic effects of the aforementioned cosmetic ingredients.

Previous studies showed that hydration levels predict skin barrier function. It is now established that intact skin barrier function is essential for healthy skin maintenance, as it serves as a first-line defense from pathogens and prevents any physical and chemical damage of the skin [20,21]. Since multiple molecular and immunological pathways are involved in the function of skin as a barrier, many cosmetic ingredients have been proposed which could impact this function [22]. Previous studies reported that niacinamide, the main ingredient in the tested product of our study, improves the skin barrier. However, the exact molecular mechanism of this effect has not been established yet, and it is assumed that niacinamide affects barrier function through ceramide synthesis [9,23]. Future studies should aim to uncover the niacinamide detailed mechanism of action, as well as the effective concentration of this ingredient in different skin conditions.

It is well known that ultraviolet radiation has detrimental effects on human skin, and our results suggest that niacinamide could help counteract these effects. It has also been described that niacinamide could affect ultraviolet radiation-induced oxidative stress which leads to melanogenesis and collagen degradation in the skin [24]. However, the majority of these findings come from in vitro or animal studies, and our clinical study could add to the body of literature in the field of photo protection and photo aging. It has also been reported that niacinamide reduces sebum production and has skin lightening properties, but these results were not observed in our study [25,26,27]. However, it should be noted that only a few of our participants reported having an oily skin type and our participants were all in the younger age group and hyperpigmentation should be evaluated in mature participants more prone to pigmentation disorders. Moreover, skin type was assessed subjectively according to available self-reported questionnaires, and this could have also impacted our results [28]. However, it is worth mentioning that the gold standard for skin type assessment has not been clearly defined or universally adopted, which leads to variations in clinical practices and research methodologies. Since niacinamide was expected to decrease sebum production and melanin levels, future studies should include higher concentrations of the ingredient and examine whether there is a dose–response relationship, i.e., whether higher concentrations lead to better effects.

The results of our study indicate that sun protection awareness levels are not as high as expected, as 60% of our participants do not use sun protection products every day. Since incidences of keratinocyte skin cancers and melanoma have been on the rise worldwide, our results suggest there is a need for education in the general population about the possible risks of sun exposure [29,30]. However, it seems that public health initiatives alone are not enough to lead to a better health outcome and national legislation and guidelines on this matter should also be introduced. For instance, Australia is a great example as it is the only country which has banned tanning beds, a well-known source of harmful ultraviolet radiation. Furthermore, Australia also introduced sales tax exemptions for sunscreen products, which could ensure greater likelihood that the general population will buy them [31]. It is worth mentioning that sun protection also includes measurements such as protecting oneself in the shade, especially between 10:00 a.m. and 4:00 p.m. and wearing clothes which could protect oneself from sun. Furthermore, recent trends show a rise in consumers’ attention towards sunscreens from natural origin and innovations in this field could be expected in the future [32,33].

To our knowledge, this is one of the first studies in which the topical application of Sr-Spider Polypeptide-1 and postbiotic Leuconostoc/Radish Root Ferment Lysate Filtrate has been performed and evaluated. Since the scientific literature on this naturally derived peptide is sparse, future studies should further investigate its effects. Furthermore, Radish Root Ferment Lysate Filtrate could have influenced the skin microbiome of the study participants, but this goes beyond the scope of our study and should be evaluated in rigorous clinical trials. Results from previous studies suggest that postbiotics, in general, may improve the skin’s barrier function, and this effect should be evaluated in both healthy and diseased skin in future randomized controlled trials [34].

Our study has several limitations. Firstly, it was conducted in a single center and in a small sample size. Furthermore, included participants were of a younger age and only Caucasians, the majority of whom did not use sun protection, which may have impacted our results, as the use of sun protection is a standard practice in many skincare regimens. Future studies should evaluate the use of niacinamide in a wider age group of participants to confirm possible effects, especially on photo-aged skin. However, even with these limitations, the tested product showed promise in skin barrier recovery and erythema reduction and further studies should confirm these results in a larger sample size.

## 5. Conclusions

Niacinamide is a notable skincare ingredient, widely recognized for its potential effects on the skin barrier, melanin synthesis, and sebum production. However, our study did not observe significant changes in melanin or sebum levels after one month of niacinamide application. The results of our study demonstrated a significant increase in skin hydration and a reduction in skin erythema following one month of niacinamide serum use. These outcomes were particularly evident in participants who did not utilize sun protection. Ultraviolet radiation is well-documented for its harmful effects on human skin, and our findings suggest that niacinamide may help mitigate the adverse impacts of oxidative stress or barrier disruption. Nevertheless, further research with a larger sample size and extended study duration is necessary to validate these results.

## Figures and Tables

**Table 1:** Demographic characteristics of study participants.

Characteristic	N (%)
Gender	
Male	20 (80.0)
Female	5 (20.0)
Smoker	
Yes	10 (40.0)
No	15 (60.0)
Daily use of the sun protection	
Yes	10 (40.0)
No	15 (60.0)
Skin type	
Oily	7 (28.0)
Mixed	6 (24.0)
Normal	6 (24.0)
Dry	6 (24.0)
Median age (range)	24 (21–28)

## Data Availability

Data are available on reasonable request to the corresponding author.

## Supplementary Materials

The following supporting information can be downloaded at: https://www.mdpi.com/article/10.3390/life14121677/s1, Serum ingredients.
