# Supplementary material for: Pilot Study on the Effects of a Cosmetic Serum Containing Niacinamide, Postbiotics and Peptides on Facial Skin in Healthy Participants: A Randomized Controlled Trial
Source: Life (Basel). 2024 Dec 18;14(12):1677. doi: 10.3390/life14121677 (PMC11727686; doi:10.3390/life14121677)
Supplement: Supplementary file 1 [file life-14-01677-s001.zip › life-3339936-supplementary.pdf]

**Serum ingredients***
1. Aqua
2. Glycerin
3. Niacinamide 5%
4. Panthenol
5. Sodium Hyaluronate
6. Vaccinium Macrocarpon Fruit Extract
7. Citric Acid
8. Collagen Amino Acids
9. Glyceryl Glucoside
10. Gluconolactone
11. Leuconostoc/radish Root Ferment Lysate Filtrate
12. Sr-spider Polypeptide-1
13. 1,2-hexanediol,
14. Caprylyl Glycol
15. Sodium Benzoate
16. Benzyl Alcohol,
17. Dehydroacetic Acid

* descending order according to ingredients concentration in the serum/cosmetic formulation
